# Methionine overaccumulation reinforces heterochromatic non-CG hypermethylation and transposon silencing in Arabidopsis

**DOI:** 10.1093/plphys/kiag503

**Published:** 2026-07-27

**Authors:** Yonatan Yerushalmy, Michal Lieberman-Lazarovich, Devasantosh Mohanty, Ron Mittler, Rachel Amir, Yael Hacham

**Affiliations:** Department of Biotechnology, Faculty of Sciences and Technology, Tel-Hai University of Kiryat Shmona and the Galilee, Upper Galilee 1220800, Israel; Faculty of Biology, Technion-Israel Institute of Technology, Haifa 3200004, Israel; Institute of Plant Sciences, Agricultural Research Organization—Volcani Institute, RishonLeZion 7505101, Israel; Division of Plant Science and Technology, College of Agriculture Food and Natural Resources, Christopher S. Bond Life Sciences Center, University of Missouri, 1201 Rollins St., Columbia, MO 65211, United States; Department of Biotechnology, Faculty of Sciences and Technology, Tel-Hai University of Kiryat Shmona and the Galilee, Upper Galilee 1220800, Israel; Division of Plant Science and Technology, College of Agriculture Food and Natural Resources, Christopher S. Bond Life Sciences Center, University of Missouri, 1201 Rollins St., Columbia, MO 65211, United States; Department of Biotechnology, Faculty of Sciences and Technology, Tel-Hai University of Kiryat Shmona and the Galilee, Upper Galilee 1220800, Israel; Migal, Galilee Technology Center, P.O. Box 831, Kiryat Shmona 1101602, Israel; Department of Biotechnology, Faculty of Sciences and Technology, Tel-Hai University of Kiryat Shmona and the Galilee, Upper Galilee 1220800, Israel; Migal, Galilee Technology Center, P.O. Box 831, Kiryat Shmona 1101602, Israel

## Abstract

Methionine is a key sulfur-containing amino acid and the precursor of *S*-adenosylmethionine, the universal methyl donor for DNA and histone methylation. While reduced *S*-adenosylmethionine availability is known to cause DNA hypomethylation, the effects of elevated methionine/*S*-adenosylmethionine remain poorly understood. Here, we addressed the effect of elevated methionine using the *mto1* mutant of *Arabidopsis thaliana*, which over-accumulates methionine and *S*-adenosylmethionine due to a mutation in cystathionine γ-synthase, the first committed enzyme of methionine biosynthesis. Whole-genome bisulfite sequencing analysis revealed widespread hypermethylation in *mto1*, particularly in non-CG contexts (CHG and CHH), with the strongest changes found in pericentromeric heterochromatin. Hypermethylation was concentrated within transposable elements (TEs), especially from the Gypsy and Copia superfamilies. Transcriptome profiling showed that TE-genes were broadly downregulated, consistent with reinforced TE silencing due to hypermethylation. When the expression level of *ONSEN* (Copia78) was examined under heat stress, it was found to be significantly lower than in the wild type. In addition, compared to wild type, *mto1* showed significantly greater reactive oxygen species accumulation. These findings suggest a link between elevated methionine levels and altered stress responses. Despite the observed epigenetic changes, the expression of core DNA methyltransferases and demethylases genes was essentially unchanged, suggesting that increased *S*-adenosylmethionine availability enhances enzymatic activity rather than gene expression. Together, these findings demonstrate that an excess of methionine/*S*-adenosylmethionine reinforces heterochromatic DNA methylation and transposon silencing, providing new insights into the connection between amino acid metabolism and epigenetic regulation in plants.

## Introduction

Methionine (Met) is a sulfur-containing amino acid that serves as a fundamental building block for protein synthesis. Its primary metabolic product, *S*-adenosylmethionine (SAM), is a crucial metabolite in plants, required for basic cellular processes such as ethylene, polyamines, vitamins, and phytosiderophores biosynthesis (Amir 2010; Ouyang et al. 2020). SAM also functions as the primary methyl group donor in the synthesis of numerous secondary metabolites, including lipids, alkaloids, and cell wall-related compounds, and is indispensable for the methylation of DNA, RNA, and histone tails (Sauter et al. 2013; Ouyang et al. 2020).

In *Arabidopsis thaliana*, SAM is synthesized by 4 SAM-synthetase enzymes (Meng et al. 2018). However, previous studies have suggested that SAM synthetase expression is not the primary factor controlling SAM content (Ranocha et al. 2001). Instead, elevated Met levels directly contribute to increased SAM accumulation (Inaba et al. 1994), with 52% to 80% of Met typically converted into SAM, and the remainder primarily incorporated into proteins (Giovanelli 1987; Ranocha et al. 2001). Given the central role of SAM, Met biosynthesis is tightly regulated, in part through a feedback inhibition mechanism whereby SAM represses the transcript levels of cystathionine γ-synthase (CGS), the first committed and rate-limiting enzyme in the Met biosynthesis pathway (Onouchi et al. 2005).

Overexpression of Arabidopsis CGS (*AtCGS*) in transgenic tobacco, soybean, potato, and Arabidopsis has been shown to substantially increase Met levels (reviewed by Amir et al. 2019). Furthermore, a modified variant of AtCGS (AtD-CGS), which lacks a 30-amino-acid regulatory N-terminal domain, escapes feedback inhibition by SAM and results in even greater Met accumulation (Onouchi et al. 2005; Hacham et al. 2006). When expressed under a seed-specific promoter, *AtD-CGS* leads to higher Met content in transgenic soybean, tobacco, and Arabidopsis seeds compared to wild-type (WT) controls (Matityahu et al. 2013; Song et al. 2013; Cohen et al. 2014, 2016). Unexpectedly, these seeds also accumulate higher levels of soluble metabolites, such as sugars, polyols, and amino acids, along with increased levels of starch and total protein, thereby improving their nutritional value.

To further understand this metabolic phenotype, seed-specific *AtD-CGS*-expressing Arabidopsis lines (SSE plants) were analyzed (Cohen et al. 2014). Transcriptomic analysis of both leaves and seeds in SSE plants revealed marked changes in the expression of genes associated with DNA and histone methylation (Girija et al. 2023). Increased DNA methylation levels were subsequently validated using methylation-sensitive restriction enzymes and colorimetric assays (Girija et al. 2023). The results showed that leaf DNA is hypermethylated (Girija et al. 2023). These findings framed our central hypothesis: Elevated Met levels lead to increased SAM levels, which, in turn, induce changes in DNA methylation and thereby might alter gene expression related to metabolism.

The main aim of this study was to test whether high Met levels are associated with DNA hypermethylation. Several studies have shown that low Met leads to hypomethylation (Li et al. 2011; Groth et al. 2016; Meng et al. 2018; Yan et al. 2019), but the impact of higher Met on the methylome remained largely unknown. To this end, we investigated the *methionine-overaccumulation 1* (*mto1*) mutant, which harbors a mutation in the *AtCGS* gene that reduces feedback regulation by SAM and leads to elevated Met and SAM levels (Inaba et al. 1994; Onouchi et al. 2005). Through integrative methylome and transcriptome analyses, we demonstrate that *mto1* exhibits widespread DNA hypermethylation, particularly in non-CG contexts located at heterochromatic regions. Notably, this hypermethylation predominantly affects specific transposable element (TE) superfamilies and is associated with downregulating TE-associated genes (TEGs). Hypermethylation of TEs has been shown to reduce their expression. Since TE could be activated under stress conditions (Ito et al. 2016; Deneweth et al. 2022), TE hypermethylation may alter the plant’s response to stress. To test this assumption, we examined ONSEN retrotransposon expression, which is activated by heat stress (Ito et al. 2016). When exposed to heat stress, the *mto1* mutant showed significantly lower *ONSEN* expression than WT, consistent with the observed hypermethylation. In addition, we found that, in response to heat stress, *mto1* accumulates significantly higher levels of reactive oxygen species (ROS) than WT, indicating an altered response to this stress. Our findings, therefore, suggest that elevated Met levels contribute to DNA hypermethylation and the epigenetic silencing of TEs, revealing a novel connection between Met biosynthesis and epigenetic regulation.

## Results

### Met content is elevated in the *mto1* mutant

The primary aim of this study was to determine whether elevated Met levels are associated with increased DNA methylation. To address this, we utilized *mto1*, which is known to accumulate higher levels of Met and SAM (Inaba et al. 1994). To confirm that these metabolites remain elevated under our growth conditions, we conducted liquid chromatography-mass spectrometry (LC-MS/MS) analysis of leaves from 21-day-old plants. The results confirmed significantly elevated levels of Met, SAM, and *S*-adenosylhomocysteine (SAH) in *mto1* compared to WT plants, with fold changes of 11.7, 2.4, and 5.6, respectively (Figure S1), consistent with previous findings (Inaba et al. 1994). The SAM/SAH ratio, known as a “methylation index” (Groth et al. 2016; Meng et al. 2018), was 12.2 ± 2.6 and 31.5 ± 6.7 in *mto1* and WT, respectively. Despite these changes, the *mto1* mutant displayed no marked morphological differences from WT under our experimental conditions.

### *Mto1* exhibits increased DNA methylation predominantly in non-CG contexts

To examine the effect of elevated Met/SAM on DNA methylation, we first measured total 5-methylcytosine (5-mC) levels using the MethylFlash Global DNA Methylation (5-mC) ELISA Easy Kit (EpiGentek). *mto1* leaves showed an ∼8% increase in global 5-mC relative to WT (Figure S2).

To characterize the DNA methylation pattern at a single-nucleotide resolution, we performed whole-genome bisulfite sequencing (WGBS) on genomic DNA from 21-day-old *mto1* and WT leaves. The average mapping rate was 71.4%. The bisulfite conversion was calculated from the chloroplast genome, with a rate of more than 99% in all samples (Table S1). As expected, methylation differed by sequence context. In WT, average methylation levels were 25.82% for CG, 6.84% for CHG, and 2.26% for CHH, consistent with previous reports (Cokus et al. 2008; Feng et al. 2020; Ni et al. 2021), while in *mto1* there was a slight increase to 25.95%, 8.24%, and 2.54%, respectively (Table S2). In both genotypes, methylation was lower in euchromatin than in heterochromatin, as expected (Table S2; Figure S3). The largest difference between *mto1* and WT was observed in heterochromatin (Figure S3), mainly due to a significant increase in CHG methylation from 21.7% to 26.7%, whereas increases in CG and CHH methylation were not significant. No significant changes were detected in euchromatin (Table S2).

**Figure 1:**
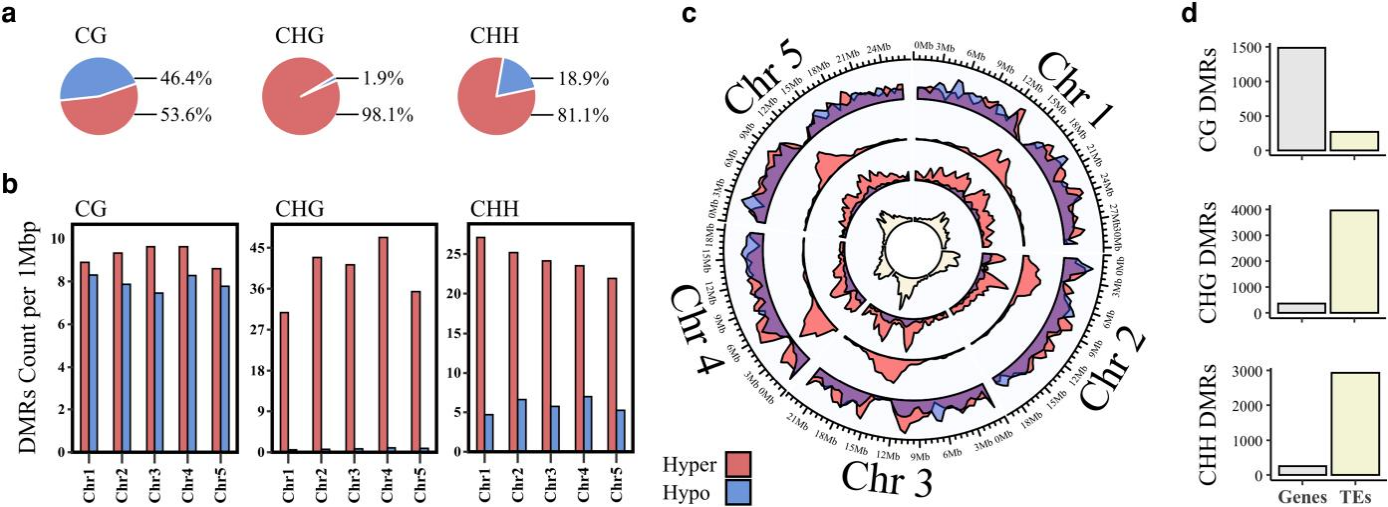
DMRs hypermethylation and hypomethylation between *mto1* and WT. a) Pie charts representing the DMRs proportion that were in hyper (red) and hypo (blue) state in the 3 contexts; b) the total numbers of DMRs that were in a state of hyper- or hypo-methylation in each of the 5 chromosomes in all contexts of methylation. DMR was counted per 1 Mbp; c) Circos plots visualizing the densities of DMRs across all the chromosomes in CG, CHG, and CHH contexts (from outer to inner circles, respectively). Red indicates hypermethylated regions, blue denotes hypomethylated regions, and purple highlights areas overlapping hypermethylated and hypomethylated DMRs. In the innermost circle, the density of TEs is shown in bright yellow and marks the pericentromeric heterochromatin regions; d) count of DMRs in CG, CHG, and CHH contexts, overlapped with coding genes and TEs.

We next defined differentially methylated regions (DMRs) using 100-bp windows and beta regression analysis (*P*_adj_ < 0.05). DMR-based analysis was used because it reduces single-site noise and better links methylation changes to functional genomic regions such as promoters, gene bodies, and TEs (Catoni et al. 2018). In total, 10,312 DMRs were detected, corresponding to 9,914 unique regions. Most DMRs occurred in the CHG context (4,679), followed by CHH (3,594) and CG (2,039). While CG-DMRs were nearly balanced between hypermethylation and hypomethylation, CHG- and CHH-DMRs were predominantly hypermethylated (Fig. 1a). After normalization per megabase, hyper-DMRs outnumbered hypo-DMRs in all contexts and across all chromosomes, with the strongest effect in non-CG contexts (Fig. 1b).

Although CG showed the highest overall methylation levels (Table S2), differences between *mto1* and WT were relatively small, likely because CG methylation is already high in WT (Figure S3) (Stroud et al. 2014). CG-DMRs were broadly distributed in the heterochromatin, pericentromeric, and euchromatic regions, but were mainly excluded from centromeric regions (Fig. 1c). In contrast, CHG-DMRs were strongly enriched in heterochromatin, especially in centromeric and pericentromeric regions, while CHH-DMRs were also concentrated in pericentromeric regions but were more diffused along chromosome arms (Fig. 1c).

To validate these patterns, we compared *mto1* with published methylation-deficient Arabidopsis mutants. Regions that were hypermethylated in *mto1* showed the opposite trend in *cmt2*, *cmt3*, *met1*, and *ddm1* mutants (Stroud et al. 2014), indicating that methylation changes in *mto1* reflect bona fide DNA methylation pathways (Figure S4). Overall, CG-DMRs included both hypermethylated and hypomethylated regions, whereas CHG- and CHH-DMRs were preferentially enriched in TE-dense heterochromatic regions surrounding the centromeres (Fig. 1a–d).

### Expression levels of DNA methyltransferase and demethylase genes

The *mto1* mutant displays pronounced hypermethylation relative to WT, particularly in non-CG contexts within heterochromatic regions and TEs (Fig. 1). In contrast, CG-context DMRs were predominantly enriched within gene bodies. To evaluate whether this hypermethylation could be attributed to increased expression of DNA methyltransferase (MT) genes, we performed RNA sequencing (RNA-seq) analysis on the same samples used for the WGBS analysis. The transcript levels of key DNA MT genes, as well as those of DNA demethylase, were examined. Expression analysis of 5 major MTs (methyltransferase 1 [MET1]; chromomethylase 2 [CMT2]; CMT3; domains rearranged methyltransferases 2 [DRM2]; and DMR3) revealed no significant differences between *mto1* and WT (Figure S5; Table S3), suggesting that the hypermethylation phenotype is not due to transcriptional upregulation of these enzymes. We further assessed whether transcript levels of DNA demethylases, which actively remove methylation marks, were affected in *mto1*. Among the 4 principal demethylase genes examined (DEMETER [DEM] and DEMETER-LIKE 1-3 [DML1-3]), 3 (DEM, DML1 [also named ROS1], and DML2) showed no substantial change, whereas DML3 expression was significantly downregulated in *mto1* (Figures S5 and S6; Table S3). This reduction in DML3 may contribute to the observed accumulation of non-CG methylation. These results suggest that DNA hypermethylation is not closely associated with gene expression levels and may instead be linked to altered MT protein activity driven by elevated SAM levels.

### Elevated Met/SAM levels in *mto1* lead to increased DNA methylation in gene bodies and flanking regions

**Figure 2:**
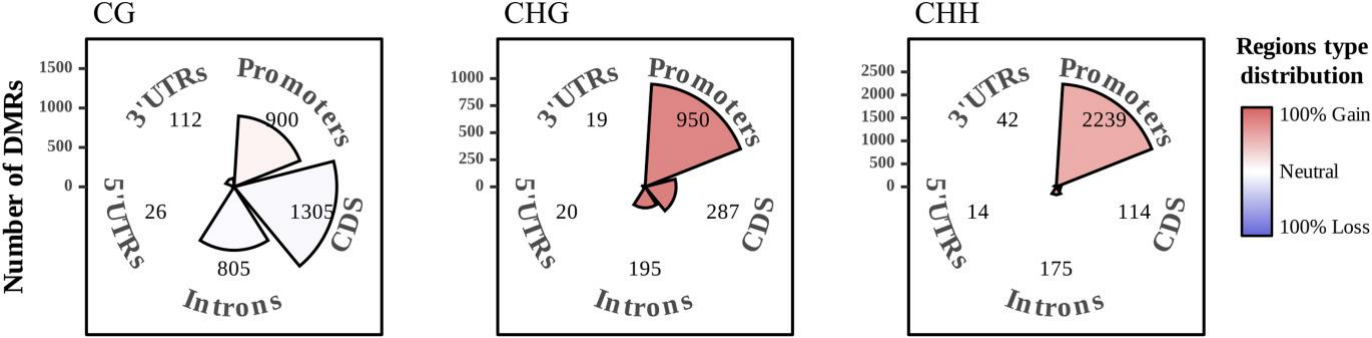
Bar plot showing differentially methylated regions (DMRs) overlapping with promoters and genic features in CG, CHG, and CHH methylation contexts in *mto1* vs wild type. The numbers displayed on the bars indicate the count of DMRs associated with each annotation. The color gradient reflects DMR counts status: white represents a 50% split between hypermethylation and hypomethylation, while gradients extend to red for 100% hypermethylation and blue for 100% hypomethylation.

Changes in DNA methylation in the euchromatic regions of *mto1* (Fig. 1c) prompted further investigation into the effects of Met/SAM accumulation on gene body methylation (GbM) and its potential impact on gene expression. We first quantified the number of DMRs and the proportion of hypermethylated DMRs within gene bodies, promoters, and genic regions between *mto1* and WT (Table S4; Fig. 2). The number of DMRs in gene bodies was markedly higher in the CG context than in the CHG/CHH. In contrast, non-CG DMRs were more enriched in the promoter regions than in gene bodies, particularly in the CHH context. Most CHG-DMRs (89% to 100%) and CHH-DMRs (78% to 86%) were hypermethylated, whereas CG-DMRs showed lower levels of hypermethylation (47% to 73%) (Table S5; Fig. 2).

### DNA methylation changes in gene bodies and flanking regions show limited overlap with DEGs

RNA-seq analysis detected 23,135 genes, of which 5,874 (25%) were significantly differentially expressed in *mto1* compared to WT. These included 11% upregulated and 14% downregulated genes. The RNA-seq results were validated using reverse transcription quantitative PCR (RT-qPCR) analysis (Figure S6). To test whether these changes were associated with changes in DNA methylation, we examined the overlap between differentially expressed genes (DEGs) and DMRs. Only a limited fraction of DEGs overlapped with DMRs: 9% contained promoter DMRs and 7% contained gene-body DMRs (Tables S4, S5, and S8). In total, 506 DEGs carried promoter DMRs, 388 contained gene-body DMRs, and 58 overlapped both categories (Text S1).

Non-CG promoter methylation is often linked to transcriptional silencing (Wang and Baulcombe 2020). In promoter regions of *mto1,* CG DMRs were both hypomethylated and hypermethylated, whereas CHG DMRs were predominantly hypermethylated. In both contexts, these methylation changes showed no consistent association with gene expression, as both up regulated and downregulated genes were observed (Tables S7 and S8). In contrast, CHH DMRs were hypermethylated by more than 2-fold, with modest enrichment for downregulated genes (Table S8). CG DMRs included both hypomethylated and hypermethylated sites, with no clear association with expression, whereas non-CG DMRs, which were mostly hypermethylated, were linked with downregulation of gene expression.

To further assess this relationship, we modeled the association between methylation levels across promoters and other genic features, as well as DEG expression, using a negative binomial regression model (Figures S7 and S8; Table S6). Similar patterns were observed in *mto1* and WT, with both positive and negative correlations depending on the locus. The negative binomial fits captured consistent trends across all contexts (Figure S7; Text S1). Overall, these results indicate that DNA methylation changes in *mto1* show only limited associations with differential gene expression.

### Elevated Met/SAM levels promote non-CG hypermethylation of retrotransposons

**Figure 3:**
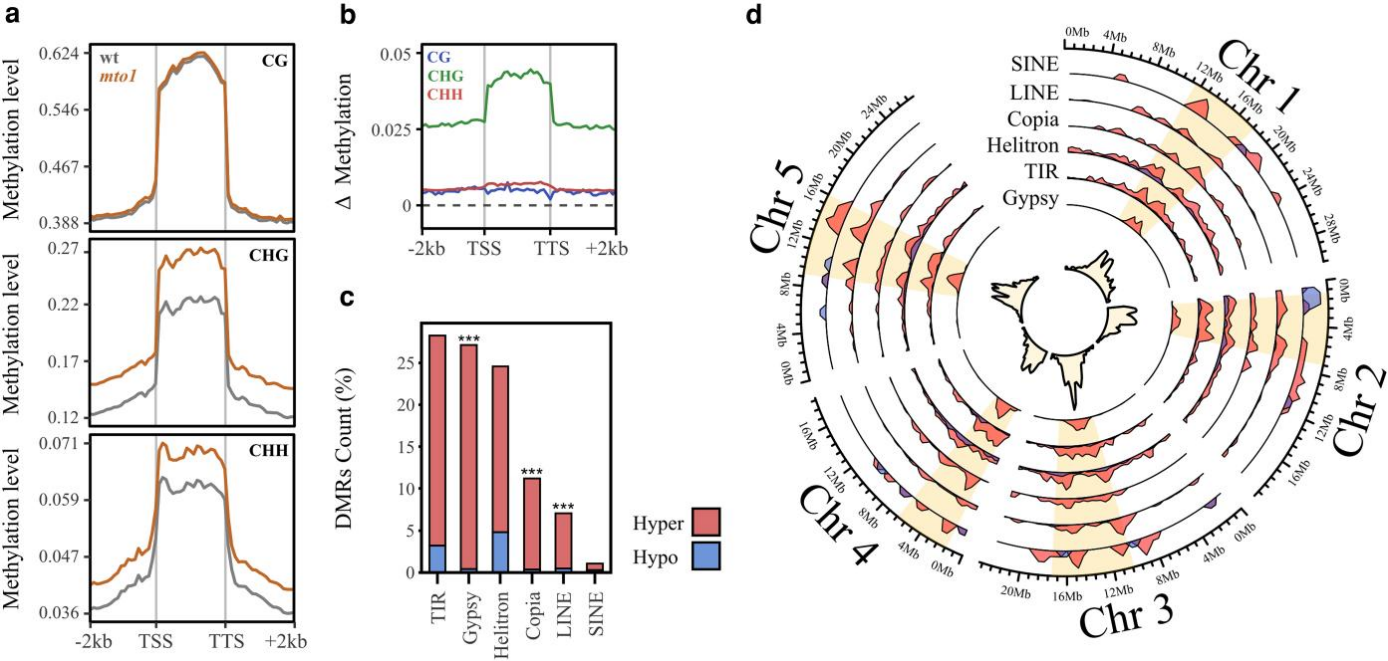
DNA methylation dynamics in transposable elements (TEs). a) Metaplots of DNA methylation in CG, CHG, and CHH contexts in *mto1* (orange) and WT (gray); b) DNA methylation difference (Δ, *mto1* vs WT) across TEs and their 2 kb upstream/downstream flanking regions. The methylation levels are plotted using mean values across 20 equally sized bins within each region; c) distribution of DMRs among major TE superfamilies (Gypsy, Copia, LINE, SINE, TIR, Helitron). The *y* axis indicates each superfamily's proportion of all TE-overlapping DMRs. Enrichment of TE copies (≥1 DMR) was tested against all other TEs using a 1-sided Fisher's exact test. Significance: *P < 0.05; **P < 0.01; ***P < 0.001; (d) Circos plot shows the chromosomal distribution of TE-overlapping DMRs from the 6 main superfamilies. Red, hypermethylated DMRs; blue, hypomethylated DMRs; purple, overlapping hypermethylated and hypomethylated regions. The yellow highlights pericentromeric heterochromatin, with TE density shown in the innermost circle. Abbreviations: TSS, transcription start site; TTS, transcription termination site.

DNA methylation is essential for genome stability, mainly through TE silencing (Quesneville 2020; Ito 2022). To examine TE methylation in *mto1*, we generated metaplots for all annotated TEs (from the TAIR10 database) and their 2-kb flanking regions (Fig. 3a, b). As in WT (Law and Jacobsen 2010), CG methylation was the most abundant methylation context, but it changed was minor in *mto1* (Fig. 3a). In contrast, CHG methylation was strongly elevated (Fig. 3a, b). In addition, CHH methylation showed changes comparable to those of CG, albeit slightly higher (Fig. 3b). Most TE-associated DMRs occurred in non-CG contexts, with 3,956 CHG-DMRs and 2,933 CHH-DMRs vs only 265 CG-DMRs (Fig. 3a, b; Tables S9 and S10).

Overall, 90.4% of TE-DMRs were hypermethylated (Table S9 and S10), with most TE-overlapping DMRs mapped to TIR, Gypsy, and Helitron elements (together representing 79.9% of all TE-DMRs) (Table S10 and S11; Figure S10). To identify which superfamilies were most strongly affected, we assessed the fraction of elements carrying ≥1 DMR (Table S12). A 1-sided Fisher's exact test showed significant enrichment of Gypsy, Copia, and LINE elements in *mto1* (Fig. 3c; Tables S11 and S12), with DMRs in these superfamilies being hypermethylated. At the family level, several lineages within the Copia, Gypsy, and LINE superfamilies were specifically enriched for DMRs (Figure S10). These include the heat-responsive element ONSEN (one of the Copia78 family), for which 7 of the 8 reference copies were hypermethylated. Also, members of the ATHILA family-related Gypsy elements (which comprised 3% of the genome) were specifically enriched (Figure S10; Table S13).

Gypsy elements were mainly enriched in centromeric and pericentromeric regions (Quesneville 2020; Ito 2022). In contrast, other TE groups were distributed more broadly along chromosome arms and heterochromatin boundaries, where they may influence gene expression in euchromatic regions (Fig. 3d). Consistent with previous reports (Thao et al. 2014), retrotransposon length was positively correlated with proximity to adjacent heterochromatin regions. In *mto1*, CHG methylation differences relative to WT increased with TE length, with clear hypermethylation in larger elements (Figure S11b), and declined with distance from the centromere (Figure S11d). At the same time, CG and CHH showed weaker size- and distance-dependent effects (Figure S11a–c). When TEs were classified by size as long (>4 Kbp) or short (<0.5 Kbp) elements, the differences in methylation between *mto1* and WT were consistently greater for long TEs, and their 2-kb flanking regions compared with short elements (Figure S12a). This effect was observed across all sequence contexts, with the most substantial increase in CHG, followed by CHH and CG (Figure S12a). Within CHG sites, retrotransposons, particularly Gypsy, Copia, and LINE elements, which showed hypermethylation, were generally longer and enriched in heterochromatin (Figure S12b). In contrast, Helitron transposons, which are typically shorter and located in euchromatin, displayed a weaker effect (Figure S12b). Together, these findings indicate that elevated Met/SAM levels in *mto1* promote hypermethylation preferably in retrotransposons, suggesting that the changes are nonrandom and target specific TE subfamilies.

### Hypermethylated DMRs suppress TEs gene expression in *mto1*

**Figure 4:**
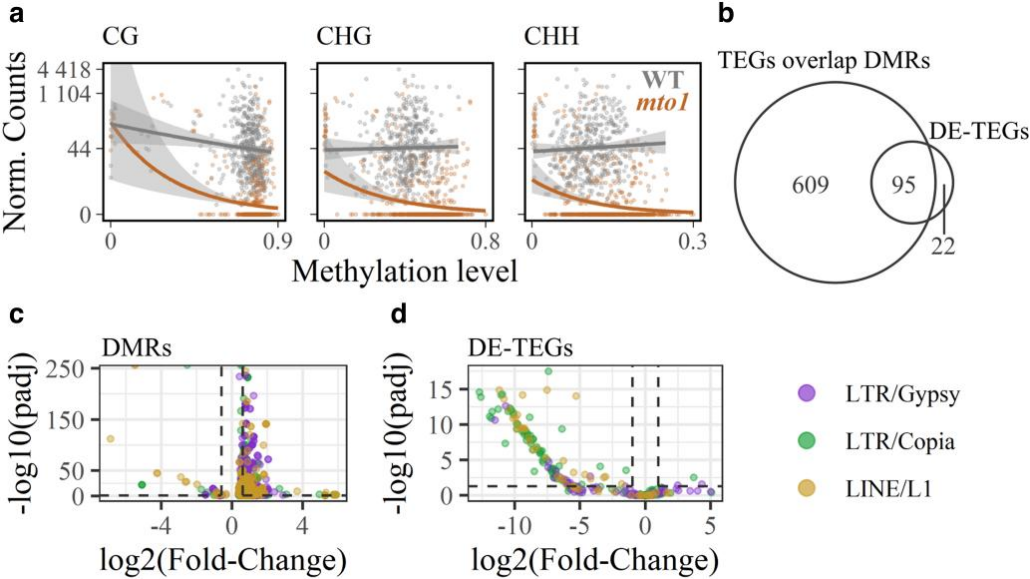
The link between DMRs and transposable element genes (TEGs) expression. a) Relationship between average DNA methylation and normalized gene expression count in *mto1* (orange) and wild type (WT, gray) for each context. Each point represents a single gene, and smooth curves depict negative binomial model fits. The *y* axis is a log1p-scaled. Model *P*-values are provided in Table S6; b) Venn diagram showing the overlap between unique TEGs containing at least 1 DMR and DE-TEGs (*P*_adj_ < 0.05). The numbers indicate unique gene IDs in each section of the diagram; c, d) volcano plot illustrating the log_2_ fold-change and adjusted *P*-values (*mto1* vs WT) for DMRs within TEGs and DE-TEGs. The data represent 3 retrotransposon superfamilies: Gypsy, Copia, and LINE; TEG lists are based on Tables S12 and S13.

To assess the impact of DMRs on the expression of Gypsy, Copia, and LINE elements, we examined the expression of TEGs, which include protein-coding sequences required for transposon mobilization, as well as transposon-derived pseudogenes (Feschotte and Pritham 2007). TEGs hypermethylation in the non-CG context was correlated with reduced TEGs expression (Fig. 4a). Although CG methylation was also negatively correlated with TEGs expression (Fig. 4a), this trend likely reflects its co-occurrence with CHG and CHH methylation at highly methylated TE loci, rather than an independent CG-specific effect. Notably, the major genotype-dependent methylation gain in *mto1* occurred in non-CG contexts, particularly CHG, indicating that increased non-CG methylation is the dominant change associated with reinforced TEG silencing.

In total, 1,480 DMRs were mapped to 704 distinct copies of Gypsy, Copia, and LINE TEGs (Fig. 4b; Tables S12 and S13), of which 98.4% were hypermethylated in *mto1* relative to WT (Fig. 4c). Among these TEGs, 117 were differentially expressed (DE-TEGs; *P*_adj_ < 0.05), and the vast majority (114; 97.4%) were downregulated (Fig. 4b–d; Table S11). Most DMRs associated with DE-TEGs occurred in non-CG contexts (Figure S3; Table S13). Notably, 95 of the 117 DE-TEGs (81%) overlapped with DMRs, and 94 out of the 95 DE-TEGs (98.9%) were DMR-hypermethylated in non-CG sites (Tables S11–S13). Consistent with stable TE silencing in WT (Daron and Slotkin 2017) and the enhanced hypermethylation observed in *mto1*, no evidence of transposition activity was detected in either genotype, as assessed by analysis of unmapped reads across the DE-TEGs, as expected. Together, these findings strongly suggest that the hypermethylation observed in *mto1*, particularly in non-CG context, contributes to the transcriptional repression of Gypsy, Copia, and LINE retrotransposons (snapshots of 2 example retrotransposons are shown in Figure S14). This effect is especially pronounced in pericentromeric regions, underscoring the critical role of DNA methylation in restricting TE activity.

### *ONSEN* shows lower expression in *mto1* during heat stress

**Figure 5:**
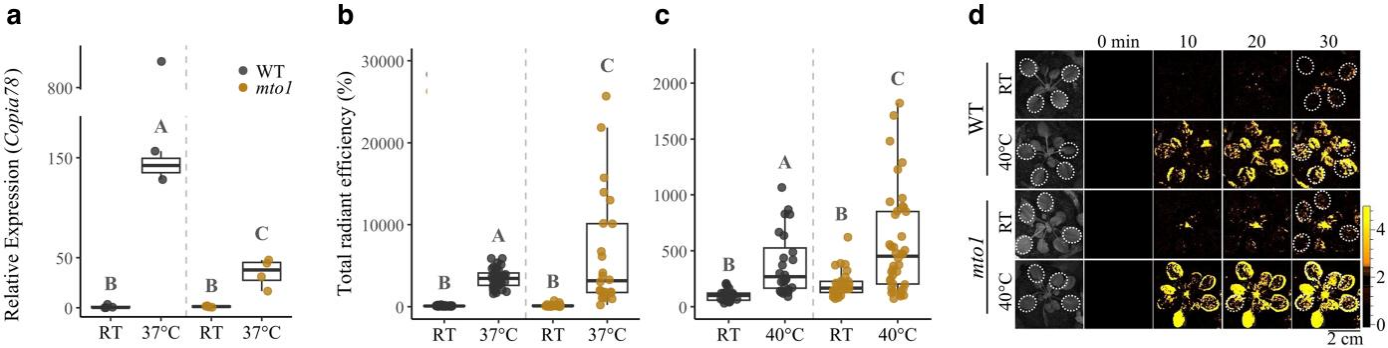
The effect of heat stress on the expression level of *ONSEN* (Copia78), and on reactive oxygen species (ROS) accumulation in WT and *mto1*. a) Relative *ONSEN* transcript levels were quantified by RT-qPCR in WT and *mto1* plants. Samples were obtained from 14-day-old plants grown at room temperature (RT) and subjected to control or a heat-stress treatment at 40 °C for 10 min. Expression values were normalized to an internal reference gene of *PP2A-3* using the 2^−ΔΔCt^ method relative to the control condition (WT at RT). b–d) *mto1* accumulates higher ROS levels than WT under heat stress. b and c) Quantification of ROS signals as total radiant efficiency. Each point represents 1 biological replicate; boxplots summarize the distribution. Thirty-day-old WT and *mto1* plants were exposed to 37 °C for 24 h (b), or to 40 °C for 10 min (c). Statistical significance among groups was determined by 2-way ANOVA followed by Fisher's LSD multiple-comparison test (*P* < 0.05); different letters indicate significant differences. d) Representative time-lapse ROS imaging of WT and *mto1* plants subjected to RT or 40 °C for 10 min.

ONSEN (a member of the Copia78 family) is an Long Terminal Repeat (LTR) retrotransposon that is transcriptionally activated by heat stress in Arabidopsis (Ito et al. 2016). In RNA-directed DNA methylation-defective plants, heat stress enables transgenerational ONSEN transposition, demonstrating that weakened silencing can convert expression into mobility. Stress-activated ONSEN mobilization can generate heritable genetic variation, potentially giving rise to new phenotypes through gene disruption or altered regulation of nearby genes (Ito et al. 2016). Our data show that the Copia78 family, particularly the ONSEN element, is significantly hypermethylated in *mto1* (Figure S10). We therefore used ONSEN as an example to test whether in *mto1*, hypermethylation leads to lower TEG expression. *ONSEN* transcript levels were measured in 14-day-old seedlings under control and heat-stress conditions. Under control conditions (room temperature, RT), *ONSEN* expression was low in both WT and *mto1*, with no significant difference between them (Fig. 5a). However, after heat treatment (37 °C for 24 h), *ONSEN* expression was strongly induced in WT (by 142-fold compared to RT). In contrast, the *mto1* mutant showed a much weaker induction (29-fold compared to RT; Fig. 5a). These results suggest that hypermethylation of *ONSEN* in *mto1* reduced its activation during heat stress.

### *Mto1* plants produce higher levels of ROS under heat stress conditions

The low expression of TEGs in *mto1* (Figs. 4 and 5a) suggests that the *mto1* mutant may respond differently to stress. Two independent experiments were conducted to test this possibility using 30-day-old *mto1* and WT plants grown in soil and exposed to heat stress. In the first experiment, the plants were exposed to 37 °C for 24 h, and in the second to 40 °C for 10 min. Heat stress is a known inducer of ROS production in plants (Mittler et al. 2002). ROS accumulation was measured following heat stress using whole-plant ROS imaging (Mittler et al. 2002; Mohanty et al. 2025). Heat stress resulted in enhanced ROS production in both WT and *mto1* plants, detectable as early as 10 min following the end of the heat stress treatment, relative to control untreated plants (Fig. 5b–d). However, compared with WT, heat-stress-induced ROS accumulation was significantly higher in *mto1*, suggesting that *mto1* has an altered response to this stress.

## Discussion

This study investigated the link between elevated Met and SAM levels and global DNA methylation patterns in *A. thaliana*. While prior studies using mutant lines with reduced SAM levels have demonstrated a reduction in global DNA methylation (Li et al. 2011; Groth et al. 2016; Meng et al. 2018; Yan et al. 2019), the impact of increased Met/SAM levels on the methylome has remained largely unexplored. The primary objective of this study was to determine the effect of *mto1*, a mutant with elevated Met/SAM levels, on the genome-wide levels and distribution of DNA methylation. Our results suggest that in *mto1* (i) elevated Met/SAM levels lead to widespread methylome and transcriptome changes; (ii) increased SAM availability promotes extensive DNA hypermethylation, primarily in non-CG contexts, with pronounced enrichment in pericentromeric heterochromatic regions and specific TE superfamilies (Figs. 1 and 2; in accordance with a previous study showing that overexpression of *Met synthase* is accompanied by a genome-wide increase in DNA methylation, primarily in the pericentromeric region [González and Vera 2019]); (iii) the effect on the methylome was not random, and was observed in specific TE superfamilies (mainly Gypsy, Copia, and LINE), and within, specific TE families in each superfamily (Figure S10); (iv) hypermethylation of TEGs is associated with reduced transcription levels; and (v) the *mto1* mutant showed lower *ONSEN* expression and higher ROS accumulation under heat-stress conditions suggesting an altered response to stress.

### The role of MTs in the hypermethylation phenotype of *mto1*

Despite the extensive non-CG hypermethylation in *mto1*, transcript levels of the major DNA methyltransferases (MET1, CMT2, CMT3, DRM1, and DRM2) were unchanged (Table S3; Figure S5). As CMT2, CMT3, and DDM1 are key regulators of non-CG methylation in heterochromatic pericentromeric regions, we propose that elevated Met/SAM in *mto1* enhances MT activity rather than MT gene expression, thereby contributing to hypermethylation. Reduced demethylation may also contribute to these changes, as the expression of DML3, the major demethylase of Arabidopsis that removes non-CG methylation, particularly at TEs and stress-related genes (Schumann et al. 2019), was significantly downregulated in *mto1*.

Higher levels of Met increase SAM levels (Figure S1; Inaba et al. 1994). SAM, as a substrate for many MTs, might increase their activities. SAH, a byproduct of the activity of MTs, acts as an inhibitor of MTs. Thus, the SAM/SAH ratio is considered a key methylation index (Groth et al. 2016; Meng et al. 2018). A higher ratio is favorable for methylation reactions, whereas a lower ratio indicates decreased methylation capacity (Groth et al. 2016). Although both SAM and SAH were elevated in *mto1*, the SAM/SAH ratio was lower than in WT, which would be typically associated with reduced methylation capacity. The increased DNA methylation in *mto1* cannot be easily explained by this SAM/SAH ratio alone. We suggest that elevated SAM may still enhance methylation reactions, while reduced DML3 expression may further limit methyl-group removal from cytosines. We assume that high SAH levels could also result from increased MT activity, which leads to higher substrate (SAM) levels. It may also increase, from other MTs, such as those involved in secondary metabolite synthesis, which may not be inhibited by SAH, allowing SAH elevation.

In addition to DNA methylation, MTs also modify histones. In Arabidopsis, non-CG methylation and H3K9me2 form a self-reinforcing loop: SUVH/KYP histone methyltransferases bind methylated DNA and deposit H3K9me2, while CMT proteins recognize H3K9me2 and maintain CHG/CHH methylation (Grewal 2023). This process reinforces TE silencing and chromatin compaction in heterochromatin (Grewal 2023). The effect of elevated Met on histone methylation, therefore, remains an important subject for future studies.

### High Met/SAM content affects GbM and genic features

In the *mto1* mutant, non-CG methylation is predominantly enriched in heterochromatic regions and TEs. Compared with WT, the mutant also shows non-CG methylation in the gene body and its flanking regions, albeit at relatively low levels (Fig. 2; Figure S12; Text S1). In WT plants, GbM is largely depleted of non-CG methylation. In *Arabidopsis*, this depletion is maintained in part by the activity of INCREASED BONSAI METHYLATION 1 (IBM1). This protein contained a jmjC domain that prevents the ectopic deposition of non-CG methylation, a process that is necessary for normal Arabidopsis development (Miura et al. 2009). The IBM1 was found to prevent CHG methylation in genes, but not in TEs (Miura et al. 2009). Therefore, average CHG/CHH changes across CDS/UTRs are expected to be small unless IBM1 (or the CMT3/SUVH pathway) is perturbed (Miura et al. 2009). We assume that in the *mto1* mutant, excess SAM can bypass IBM1 activity, or that another unknown process might occur.

Total CG methylation is naturally highest in pericentromeric heterochromatin, and the overall mCG level in these regions is similar between WT and *mto1* (Table S2), consistent with previous methylome studies done on the WT (Cokus et al. 2008; He et al. 2022). Because heterochromatic mCG is already near saturation in WT, additional increases in *mto1* are likely constrained. It may also be that MET1 activity in this region is low. In contrast, we observed a significant increase in CG methylation in euchromatin (Fig. 1c, d). Accordingly, while absolute CG methylation levels remained highest in pericentromeric heterochromatin, CG-context DMRs in *mto1* were enriched predominantly within gene bodies on the chromosome arms. These gene-body CG DMRs showed markedly higher methylation in *mto1* than in WT (Fig. 1d).

Total CG methylation is highest in heterochromatin and the pericentromeric region and is similar in WT and *mto1* (Table S2) (Cokus et al. 2008; He et al. 2022). Given the high levels in the WT, the CG context may be almost fully methylated in mCG, or MET1 activity in this region may be low. Although we did not detect significant changes in mCG in heterochromatin, we did observe significantly higher methylation in euchromatin (Fig. 1c, d). Thus, whereas absolute CG methylation levels remained highest in pericentromeric heterochromatin, in the *mto1* mutant, CG-context DMRs were predominantly enriched within gene bodies located on the chromosome arms, whereas absolute CG methylation levels remained highest in pericentromeric heterochromatin. The DMRs of CG in gene bodies were markedly higher in *mto1* compared to WT (Fig. 1d).

In *A. thaliana*, approximately one-third of all genes exhibit GbM, with ∼20% showing CG methylation specifically in exonic regions (Cokus et al. 2008; He et al. 2022). Although the functional significance of GbM remains under debate (Muyle et al. 2022), GbM has been proposed to contribute to multiple processes, including the regulation of gene expression, suppression of cryptic transcription, prevention of intron retention, modulation of splicing, and protection from TE insertions (Cokus et al. 2008; He et al. 2022).

In *mto1*, where Met/SAM levels are elevated, changes in GbM, including both hypermethylation and hypomethylation, did not consistently correlate with gene expression levels (Tables S2 and S8). Similarly, in the *atms1-1* mutant, which exhibits global hypomethylation due to impaired Met synthesis, GbM alterations were not associated with differential gene expression (Yan et al. 2019).

Promoter methylation is an important regulator of gene expression. In *A. thaliana*, about 5% of genes carry promoter methylation across all sequence contexts, and this is generally associated with transcriptional repression, although exceptions occur (Zhang et al. 2006; Kumar and Mohapatra 2021; He et al. 2022). In *mto1*, however, promoter CG-DMRs showed no consistent directional association with gene expression, as both upregulated and downregulated genes were observed (Table S2, Figure S6). Similarly, CHG hypermethylation did not correlate with transcriptional changes (Table S2). By contrast, CHH-DMRs were more than twice as frequent and were predominantly hypermethylated, coinciding with a modest increase in downregulated genes (Tables S2 and S9). Promoter CHH hypermethylation has also been reported under abiotic and hormonal stress conditions, suggesting greater regulatory flexibility in this context (Wang and Baulcombe 2020; López et al. 2022). Consistent with this, the relationship between promoter methylation and gene expression appears to depend on the species, condition, and locus under question (Xu et al. 2018; Wang and Baulcombe 2020; López et al. 2022).

### The effect of elevated Met/SAM TE methylation

In *mto1*, the increase in non-CG methylation in heterochromatin occurs mainly at TEs (Fig. 1), which are naturally enriched and heavily methylated (Wang and Baulcombe 2020; He et al. 2022; Muyle et al. 2022). In contrast, mutants with reduced SAM levels, such as *atms1-1* and *mat4*, show widespread hypomethylation, especially in pericentromeric regions, accompanied by TE activation (Meng et al. 2018; Yan et al. 2019), highlighting the importance of DNA methylation in TE silencing (He et al. 2022).

The TE groups most affected in *mto1* were Gypsy, Copia, and LINE (Table S12; Fig. 3c). Their nonrandom DMR distribution suggests that specific TE families are particularly susceptible to methylation changes. Among these, CHG methylation showed a higher proportion of hypermethylated DMRs than CHH (Table S11; Fig. 3b; Figure S12), likely reflecting re-establishment required for mCHH in each generation (Lee et al. 2023). TE hypermethylation in *mto1* was associated with reduced expression of TEGs (Table S1; Fig. 3; Figure S13), supporting enhanced TE silencing. As in WT under nonstress conditions (Daron and Slotkin 2017), no evidence of transposition was detected in *mto1*, either within the DE-TEG list (Table S10) or specifically for the *ONSEN* element. These findings are consistent with previous studies highlighting the critical role of non-CG methylation in TE repression (Quesneville 2020; Wang and Baulcombe 2020; Ito 2022; Muyle et al. 2022).

The ONSEN element was significantly hypermethylated in *mto1* (7 out of 8 copies contained >1 DMR), and its heat-induced expression was markedly lower than in WT (Fig. 5a), indicating reinforced silencing. Because ONSEN and other Copia elements can alter nearby stress-related genes when activated (Ito et al. 2016), their repression in *mto1* may reduce genome plasticity, particularly under stress conditions, when TE activation is normally enhanced (Ito 2022). In future studies, it would be interesting to examine how *mto1* responds to stress, such as oxidative stress and heat stress.

### ROS levels are higher in the *mto1* mutant under heat stress conditions

The reduced TE expression in *mto1* may limit transcriptional flexibility during stress. To test whether the stress response of *mto1* differs from that of WT, plants were exposed to heat stress. While the basal levels of ROS in *mto1* and WT were comparable, the experiments revealed that *mto1* accumulated more ROS than WT after both short-term (10 min) or prolonged (24 h) heat treatment (Fig. 5), indicating an altered response to heat stress.

Heat stress is known to increase ROS levels, which may play signaling or toxic roles (Mittler et al. 2002). Depending on their level and localization, ROS can therefore either activate defense and acclimation pathways that promote thermotolerance or cause cellular damage when production exceeds detoxification capacity (Mittler et al. 2002; Choudhury et al. 2017). As signaling ROS are thought to play a role during early stages of stress perception (Mittler et al. 2002), the prolonged ROS accumulation (after 24 h at 37 °C) observed in *mto1* could reflect sustained oxidative stress, although ROS-dependent signaling could also be a possible explanation (Mittler et al. 2002; Choudhury et al. 2017). As *mto1* accumulated significantly higher levels of ROS following short- or prolonged-heat stress treatments (Fig. 5b), further studies are needed to resolve the relationship between altered methylation and ROS-mediated stress signaling during heat stress.

Interestingly, plants with elevated Met/SAM levels were mostly found to be more sensitive to biotic and abiotic stress. For example, overexpression of *Met synthase* or *AtCGS*, both associated with DNA hypermethylation (González and Vera 2019; Girija et al. 2023), increased susceptibility to *Pseudomonas syringae* (González and Vera 2019), or sensitivity to oxidative stress (Hacham et al. 2024). In contrast, DNA hypomethylation or increased DNA demethylase activity has been associated with improved resistance to heat, salt, drought, and pathogens (Yu et al. 2013; Jiang et al. 2021; Lee et al. 2023). Together, these studies suggest that hypermethylation is associated with reduced tolerance to stress, whereas hypomethylation confers resistance.

## Conclusion and perspectives

This study establishes a link between elevated Met/SAM levels and increased DNA methylation, particularly in the non-CG context. The DMRs were not randomly distributed but were predominantly enriched in centromeric and pericentromeric heterochromatin, which is rich in TEs. In *mto1*, the number of methylated Class I retrotransposons was significantly higher compared to WT, coinciding with a substantial reduction in the expression of TEGs, including the heat-responsive element ONSEN, implicated in epigenetic regulation (Deneweth et al. 2022). The *mto1* mutant shows significantly higher ROS accumulation during heat stress, suggesting it is less tolerant to stress.

One potential explanation for the preferential hypermethylation of heterochromatin is related to Met metabolism. Elevated Met is known to impair plant growth and development (Hacham et al. 2002; Xiang et al. 2022). To mitigate toxicity, plants catabolize Met into volatile compounds or divert its intermediates into the biosynthesis of secondary metabolites such as glucosinolates, polyamines, *S*-methylMet, and isoleucine (Hacham et al. 2002; Cohen et al. 2014; Hacham et al. 2023). Nonetheless, the precise mechanisms of Met toxicity remain elusive. Recent findings show that increased Met biosynthesis impairs the plant's ability to cope with oxidative stress (Hacham et al. 2024). The current study suggests a mechanism for managing excess Met/SAM by directing methyl groups toward heterochromatin regions that are less essential to daily growth and development than euchromatin. This redistribution might represent an adaptive buffering strategy to protect the epigenome from high SAM content and methylation overload. However, the widespread transcriptional changes observed in *mto1* indicate that additional, yet unidentified, mechanisms may be involved.

In summary, our findings highlight the impact of elevated Met/SAM levels on epigenetic regulation in plants and suggest a novel link between primary metabolism and DNA methylation patterning. Future research should unravel the mechanisms governing the integration between SAM abundance, MTs activity, chromatin remodeling, and plant stress responses.

## Materials and methods

### Plant genotypes and growth conditions

The Arabidopsis (*A. thaliana*) mutant lines of *mto1* (CS196) were obtained from ABRC, Ohio State University, United States. *mto1* and WT (*Col-0*) plants were grown in 0.5 Murashige and Skoog media (DuShefa, Haarlem, The Netherlands) supplemented with 1% sucrose under a 16-h light/8-h dark cycle in a growth chamber (22 °C ± 2 °C). The plants were subsequently transferred to soil and placed in a growth chamber under the same light conditions at 22 °C ± 2 °C.

### Metabolite detection

Twenty-one-day-old Arabidopsis leaves were frozen in liquid nitrogen and lyophilized. The lyophilized tissues were ground to a fine powder in liquid nitrogen and kept at −80 °C until use. Met, SAM, and SAH were extracted from 50 mg of dry powder. The samples were analyzed by injecting 5 µL of the extracted solutions (100 mg/mL of aqueous solution with 0.01N HCl) into an Ultra-high performance liquid chromatography connected to a photodiode array detector (Dionex Ultimate 3000), with a reverse-phase column (Phenomenex RP-18, 100x 3.0 mm, 2.5 μm). The mobile phase consisted of (A) DDW with 0.1% formic acid and (B) acetonitrile containing 0.1% formic acid. The gradient started with 100% A for 1 min, then increased to 30% B in 7 min, then to 90% B in 2 min, and kept at 90% B for another 3 min. Phase A was returned to 100% A in 1 min, and the column was allowed to equilibrate at 100% A for 2 min before the next injection. The flow rate was 0.5 mL/min. LC-MS/MS analysis was performed with a heated electrospray ionization (HESI-II) source connected to a Q Exactive Plus Hybrid Quadrupole-Orbitrap Mass Spectrometer Thermo Scientific. ESI capillary voltage was set to 3,500 V, capillary temperature to 300 °C, gas temperature to 350 °C, and gas flow to 10 mL/min. The mass spectra (*m/z* 100 to 1,500) were acquired in positive-ion mode.

### Whole-genome bisulfite sequencing

Genomic DNA from leaves of 3 biological replicates of *mto1* and 2 of WT was extracted using a DNeasy Plant Mini Kit (Qiagen). We generated global DNA methylation profiles at single-nucleotide resolution using the Illumina Hi-Seq 2500 platform (BGI, Tech Solutions, Hong Kong). DNA quality and contamination were monitored by running samples on 1% agarose gels. From each sample, 1 μg of genomic DNA was sent to BGI (BGI, Shenzhen, China) for further preparation and analysis.

Genomic DNA was prepared for library construction by fragmenting to an average size of 200 to 350 bp, followed by blunt-ending, 3′-dA addition, and ligation of methylated adaptors. The libraries underwent bisulfite conversion using the EZ DNA Methylation-Gold Kit (ZYMO), PCR amplification, quality control, circularization, DNA nanoball formation, and sequencing on the DNBSEQ platform (DNBSEQ Technology). Raw reads were processed using SOAPnuke (Chen et al. 2018) with the following filtering parameters: removal of adaptor sequences (≥25% match, ≤2 mismatches), reads <150 bp, reads containing ≥0.1% ambiguous bases (N), reads with polyX stretches >50 bp, or reads with ≥40% of bases having Phred scores <20. After filtering, only high-quality reads (Phred+33 encoding) were retained for downstream analysis.

### Analysis of RNA-sequencing

Total RNA was isolated from the leaves of 3 *mto1* and WT plants using the Spectrum Plant Total RNA kit (Sigma) according to the manufacturer's protocol. The quality and integrity of the RNA were checked by running samples on 1% agarose gels and using a standard NanoDrop.

RNA-seq libraries were prepared at the Crown Genomics Institute of the Nancy and Stephen Grand Israel National Center for Personalized Medicine, Weizmann Institute of Science, using the INCPM-mRNA-seq. Briefly, the poly (A) fraction (mRNA) was purified from 500 ng of total input RNA, followed by fragmentation and generation of double-stranded cDNA. After Agencourt Ampure XP beads cleanup (Beckman Coulter), end repair, A base addition, adapter ligation, and PCR amplification were performed. Libraries were quantified using a Qubit (Thermo Fisher Scientific) and a TapeStation (Agilent). Sequencing was done on a Novaseq 6000 instrument (Illumina) using an S1 100 cycles kit, allocating 40 to 50 M reads per sample (paired-end sequencing).

### Reverse transcription quantitative PCR

For RT-qPCR, RNA extraction was performed as described above for RNA-sequencing. RNA samples were treated with DNase I (Promega) for 15 min. First-strand DNA was synthesized at 42 °C with 0.5 µg total RNA as a template, using Verso cDNA Kit (Thermo Scientific) with oligo(dT) primer. RT-qPCR assays were performed in the Applied Biosystems StepOnePlus system using 2 × qPCRBIO Fast qPCR SyGreen Blue (PCR BIOSYSTEMS). The conditions were set as follows: an initial polymerase activation step for 3 min at 95° followed by 42 cycles of 95 °C for 3 s, 60 °C for 30 s. Primers for the selected genes are found in Table S14. To normalize the variance among samples, the PP2A-A3 transcript level was used as an endogenous control. The values presented are the mean of 3 biological replicates, each with 3 technical replicates. Relminus delta delta Ct the $2^{-\Delta \Delta \mathrm{Ct}\;}$ method, using the WT's RT control condition as calibrator.

### Whole-plant live imaging of ROS accumulation following heat stress

Thirty-day-old WT and *mto1* were fumigated with 10 mL of 10 μM 2′,7′-Dichlorodihydrofluorescein diacetate (H_2_DCFDA; a cell-permeable ROS-specific dye; excitation/emission 480 nm/520 nm; cat. no. 4091-99-0; Sigma-Aldrich, St. Louis, Missouri, United States) solutions that were made in 10 mM phosphate buffer (pH 7.4) containing 0.002% Silwet L-77 (LEHLE seeds, Round Rock, Texas, United States) in a glass chamber (24.5 × 12.5 × 16.5)″ as previously described (Fichman et al. 2019). Post-fumigation, half of the WT and *mto1* mutant plants were exposed to heat stress at (i) 40 °C for 10 min or (ii) 37 °C for 24 h, under 100 μmol photons m^−2^ s^−1^, while the other half served as untreated control plants (Mohanty et al. 2025). Immediately after stress exposure, the heat-treated and the untreated control plants were imaged using the IVIS Lumina S5 platform using Living Image 4.7.3 software in acquisition mode (PerkinElmer, Waltham, Massachusetts, United States) for 30 min as described in Fichman et al. (2019) and Mohanty et al. (2025). Total radiant efficiency of regions of interest was measured using ImageJ as described by Mohanty et al. (2025). Each experiment was conducted at least 3 times, with at least 4 technical replicates per genotype. Data points from all experiments were pooled together to generate the final figure.

### Data analysis

All analyses used the *A. thaliana* TAIR10 genome assembly and annotations. TEs were grouped by transposition mechanism following an Arabidopsis-focused framework (Quesneville 2020): Gypsy, Copia, LINE, and Helitron were treated as separate groups; all terminal inverted repeat (TIR) superfamilies, such as En-Spm, MuDR, Harbinger, hAT, Pogo, Mariner, and Tc1 were grouped into a single “TIR” category; SINE elements annotated as Rath (RathE*_cons) were included within the SINE order; elements annotated as “Unassigned” were excluded from downstream analyses. TEGs were defined as gene models annotated with type “transposable element gene” in TAIR10. Each TEG was linked to its source TE via the GFF3 “Derives_from” attribute. To examine the transition activity, we used new nonreference TE insertions called from unmapped WGBS reads using epiTEome (default settings; ≥5 supporting split reads; evidence from both TE ends) against the TAIR10 genome and TE annotations (Daron and Slotkin 2017). Methylome WGBS reads were aligned to the TAIR10 genome using Bismark software (Krueger and Andrews 2011). Per-cytosine methylation levels were derived from Bismark cytosine calls as methylated reads/methylated reads+unmethylated reads $\frac{methylated\;reads\;}{total\;reads}$ at each covered cytosine. Average methylation (%) was the mean of these per-C values across all covered cytosines within a region per sample and summarized as mean ± SD across replicates. For meta-plots, we oriented all loci as 5′→3′, split each protein-coding gene (or TE) and the ±2 kb flanks regions into 20 length-normalized bins (60 total), computed per-bin mean methylation level for CG/CHG/CHH contexts using sites with ≥6× coverage, then averaged bin-wise across all loci. DMRs were calculated using the *DMRcaller* R package (Catoni et al. 2018) in 100-bp bins, applying minimum methylation difference thresholds of 0.4, 0.2, and 0.1 for the CG, CHG, and CHH contexts, respectively, with a ≥ 6× coverage and ≥4 cytosines per site. Enrichment tests (1-tailed) were performed by comparing the DMR counts in each group to those in its corresponding larger group, with *P*-values and scores calculated using PlanTEnrichment method (Karakülah and Suner 2017). Heterochromatin regions were defined as the pericentromeric intervals as previously define (Bi et al. 2017), on TAIR10: Chr1 ∼11.5 to 17.7 Mb; Chr2 ∼1.1 to 7.2 Mb; Chr3 ∼10.3 to 17.3 Mb; Chr4 ∼1.5 to 6.3 Mb; Chr5 ∼9.0 to 16.0 Mb; all other sequence was considered euchromatin. For RNA-seq data, clean reads were mapped to the Arabidopsis genome using RSEM (Li and Dewey 2011) to generate normalized expression values for each gene. They DEGs (*P*_adj_ < 0.05) were identified with DESeq2 (Love et al. 2014). To ensure reliable quantification, only genes with normalized read counts >10, and TEGs with >20 normalized read counts, were included in the analysis. Both DMRs and DEGs statistical tests were adjusted to the *P*-values (*P*_adj_) for multiple testing using Benjamini and Hochberg's method (Love et al. 2014; Catoni et al. 2018). Negative binomial regression was used to model normalized gene expression read counts as a function of average methylation level across gene bodies and promoter regions while accounting for genotype differences (*mto1* vs WT). All data visualizations and statistical analyses were conducted using R (R Core Team 2018). The R scripts used for the various analyses in this study, and the methylome analysis report (including quality control, downstream analyses, and visualization) are available on GitHub at https://yo-yerush.github.io/mto1-induced-non-CG-hypermethylation/Methylome.At_report_mto1_vs_wt.html.

## Supplementary Material

kiag503_Supplementary_Data

## Data Availability

All high-throughput sequencing data from this study are available in the NCBI BioProject database under the accession number: PRJNA1206675. Additional supporting data can be requested from the corresponding authors. The Arabidopsis reference genome (TAIR10), including gene and transposable element annotations, was obtained from The Arabidopsis Information Resource (TAIR; www.arabidopsis.org).
