# Supplementary material for: Methionine overaccumulation reinforces heterochromatic non-CG hypermethylation and transposon silencing in Arabidopsis
Source: Plant Physiol. 2026 Jul 27;201(4):kiag503. doi: 10.1093/plphys/kiag503 (PMC13464503; doi:10.1093/plphys/kiag503)
Supplement: Supplementary file 1 [file kiag503_supplementary_data.zip › Supplementary Text 1.docx]

**Supplementary Text 1- The effect of higher Met/SAM on the DNA methylation in gene bodies and flanking regions**

## **DNA methylation in gene bodies and flanking regions and the putative relation to gene expression in *mto1***

To evaluate the impact of DMRs on expression, we analyzed DEGs with at least one DMR (Tables S6-S8). Promoter methylation plays a critical role in regulating gene expression. In *A. thaliana*, approximately 5% of genes exhibit methylation in their promoter regions, occurring across all sequence contexts and typically associated with transcriptional repression (Zhang et al., 2006; Kumar and Mohapatra, 2021). However, exceptions to this trend have been reported (He et al., 2022). In the *mto1* mutant, 8.6% of total DMRs are in promoter regions, with 2.27% in the CG context and 6.86% in non-CG contexts. Next, we are interested in revealing the effect of these DMRs on gene expression. The analysis shows that 18.1% of DMR within the promoters were associated with altered gene expression.

Within genic features, DMRs in CDS, introns, 5′ UTRs, and 3′ UTRs were linked to differential expressions at rates of 22.9%, 24.1%, 27%, and 24.8%, respectively, from the total DMRs in each feature (Fig. T1; Tables S7-S8, filtered by unique genes). Overall, 55.4% of DEGs that have DMRs were downregulated (Table S8). CG-DMRs showed balanced hypo- and hypermethylation patterns, while non-CG DMRs were predominantly hypermethylated.

**Figure T1.** A Venn diagram shows the overlap between differentially expressed (DEGs) (orange) and DMRs (that contain at least one DMR) (gray) in the (A) promoters, (B) CDS, (C) introns, (D) 5’-UTRs, (E) 3’-UTRs (padj < 0.05). The numbers indicate unique gene IDs in each section of the diagram. Percentages represent the proportion of genes with overlapping DMRs and DEG compared to all genes with DMRs in the respective genomic feature. The data relies on Tables S8–S9.

Previous studies suggested that CG gene body methylation (GbM) is higher in stable housekeeping genes than in dynamically expressed genes (Williams et al., 2023). However, our analysis showed that stable genes differentially expressed in *mto1* had a CG GbM pattern similar to that of dynamic genes (Tables S7, S8).

Despite higher variability in TEG expression (indicated by a lower dispersion parameter, θ), the deviance-based R² was higher than for other gene loci (Fig. S8), suggesting that DNA methylation accounted for a substantial portion of the variation in TEG expression.
