# Supplementary material for: Methionine overaccumulation reinforces heterochromatic non-CG hypermethylation and transposon silencing in Arabidopsis
Source: Plant Physiol. 2026 Jul 27;201(4):kiag503. doi: 10.1093/plphys/kiag503 (PMC13464503; doi:10.1093/plphys/kiag503)
Supplement: Supplementary file 1 [file kiag503_supplementary_data.zip › Supplementary_Figuers_Final.docx]

**Supplementary Figures**

**Figure S1.** The levels of Met, SAM, and SAH in mto1 and WT were measured by LC-MS. Data are presented as the means ± SD of four replicates. Statistical significance compared to WT was determined using an unpaired Student’s t-test (P < 0.05), indicated by asterisks.

**Figure S2.** Change in the percentage of DNA methylation in leaves of *mto1* compared to WT plants, as obtained with MethylFlash™ Global DNA Methylation (5-mC) ELISA Easy Kit (Epigentek). The data is presented as the means ± SD of three (WT) or four (*mto1*) biological replicates taken in two technical replicates. Statistical significance compared to WT was determined using an unpaired Student’s t-test (P < 0.05), indicated by asterisks.

**Figure S3.** Genome-wide methylation levels for *mto1* (orange) and wild type (WT, gray), shown in 150-kbp sliding windows across the five chromosomes for CG, CHG, and CHH (top to bottom, respectively). The bottom track illustrates the density of transposable elements (TEs) (based on TAIR10 annotations), highlighting pericentromeric heterochromatic regions.

ראש הטופס

תחתית הטופס

**Figure S4.** Genome-wide distribution plots depicting methylation differences (Δ methylation-level) between *mto1*, various methyltransferase mutants, and wild-type (WT) Arabidopsis plants. Methylation differences are represented in 150-kbp sliding windows across all five chromosomes in CG, CHG, and CHH (top to bottom, respectively). The bottom track illustrates the density of transposable elements (based on TAIR10 annotations), highlighting pericentromeric heterochromatic regions. Data for methyltransferase mutants (WT-2, WT-3, *met1*, *cmt2*, *cmt3*, *ddm1*) were sourced from Stroud et al., 2013 (GEO accession IDs: WT-2, GSM980986; WT-3, GSM980987; *met1*, GSM981031; *cmt2*, GSM981002; *cmt3*, GSM981003; *ddm1*, GSM981009).

**Figure S5.** Bar plot showing the differential expression (log₂ fold-change; p-Value > 0.05) of five methyltransferase genes and four demethylases in *mto1* relative to wild-type. The Table is based on Table S3.

WT	*mto1*

WT	*mto1*

**Figure S6.** **Validation of the RNAseq analysis**. The transcript levels were quantified by Real time qPCR and normalized to an internal reference gene of *PP2A-A3* using the 2^-ΔΔCt^ method relative to wild type (WT). Five biological replicates were taken from 28-day-old plants. Statistical significance among plants was determined by Student's T-test (P < 0.05).

**Figure S7.** Relationship between average DNA methylation and normalized gene expression count in *mto1* (orange) and wild type (gray). Each point represents a single gene, and smooth curves depict negative-binomial model fits. The Y-axis is a log1p-scaled. Model p-values are provided in Table S12. Bar plots summarizing the dispersion parameter (θ) and deviance-based R² values are shown in Fig. S7. Abbreviations: M, methylation effect; G, genotype effect; M×G, interaction; ns, not significant; n.e., not estimable (due to separation or near-zero counts).

(A)

(B)

**Figure S8.** (A) Bar plot of the dispersion parameter (θ) from the negative binomial model fitted to each genomic feature (x‐axis) in different DNA methylation contexts (CG, CHG, CHH). Error bars represent standard errors (SE) of θ. Larger θ values indicate lower dispersion (less variance than the mean); (B) Bar plot of the  deviance-based R² ($1- \frac{residual deviance}{null deviance}$) from negative-binomial models computed for each genomic feature in the CG, CHG, and CHH contexts. Larger values indicate a better fit of the negative binomial model for that feature/context combination.

**Figure S9.** Pie chart depicting the distribution of the total TAIR10 transposable elements (Total TEs) and TE-overlapping differentially methylated regions (DMRs) across TE superfamilies, grouped by cytosine context (CG, CHG, CHH). Superfamily classification was followed by Quesneville (2020): Class II TIR elements include En-Spm, MuDR, Harbinger, hAT, Pogo, Mariner, and Tc1; while SINEs also comprise RathE. The data is based on Table S3.

**Figure S10**. Enrichment analysis comparing the proportion of TEs with DMRs in each family to the total TEs with DMRs in their respective TE group: (A) Copia, (B) Gypsy, and (C) LINE. Fisher’s p-value marked as ‘*’; ‘**’; ‘***’ for p < 0.05; p < 0.01; p < 0.001, respectively.

**Figure S11.** Methylation difference (Δ , *mto1* *vs*. WT) in transposable elements (TEs). Δ methylation versus TE size for (A) CG, (B) CHG, and (C) CHH contexts; each point is a single TE. The red line is a linear model with an SE band, and the gray dashed line represents 4Kbp-TE size, which is considered a long TE. (D) Δ methylation (per context) versus absolute distance to the centromere of the TE chromosome, averaged in 1-Mbp windows. TE size in kbp; distances in Mbp.

**Figure S12.** Metaplots of DNA methylation difference (Δ, *mto1* *vs*. WT) across (A) long (>4000bp) and short (<500bp) transposable elements (TEs), and (B) the major TE-superfamilies. The metaplots show Δ methylation levels within TEs and their 2 kb upstream/downstream flanking regions in CG, CHG, and CHH contexts (blue, green, and red, respectively). The Δ methylation levels are plotted using mean values across 20 equally sized bins within each region. Abbreviation: TSS, transcription start site; TTS, transcription termination site.

**Figure S13.** Volcano plot displaying the fold-change (*mto1* vs. WT, in log₂ scale) for DMRs within TEGs in CG, CHG, and CHH contexts from the retrotransposon superfamilies of Gypsy, Copia, and LINE. The TEGs lists rely on Table S13.

**Figure S14.** Snapshot views of methylation and expression over two transposable element genes (TEGs), (A) AT5G19097 and (B) AT4G15590. For each locus, the plot shows the full annotated TEG together with 4-kb upstream and downstream flanking regions. The reference gene model is shown in black, with internal white arrows indicating transcriptional direction. RNA-seq coverage is displayed as bars, with WT shown in gray and *mto1* in orange. Differentially methylated regions (DMRs) are plotted below the RNA-seq track, separated by sequence context (CG, CHG, and CHH). Smoothed per-replicate methylation profiles (150bp window size) are shown for CG, CHG, and CHH contexts in the bottom three tracks; WT replicates are plotted in gray tones and mto1 replicates in orange tones.
